# Metabolic analysis of early nonalcoholic fatty liver disease in humans using liquid chromatography-mass spectrometry

**DOI:** 10.1186/s12967-021-02820-7

**Published:** 2021-04-15

**Authors:** Cheng Hu, Tao Wang, Xiaoyu Zhuang, Qiaoli Sun, Xiaochun Wang, Hui Lin, Mingli Feng, Jiaqi Zhang, Qin Cao, Yuanye Jiang

**Affiliations:** 1 Experiment Center for Science and Technology, Shanghai University of Traditional Chinese Medicine, Shanghai, 201203 China; 2 Department of Gastroenterology, Putuo Hospital, Shanghai University of Traditional Chinese Medicine, 164 Lanxi Road, Shanghai, 200062 China; 3 Institute of Digestive Diseases, Longhua Hospital, Shanghai University of Traditional Chinese Medicine, Shanghai, 200032 China; 4 Shanghai TCM-Integrated Institute of Vascular Anomalies, Shanghai TCM-Integrated Hospital, Shanghai University of Traditional Chinese Medicine, Shanghai, 200082 China

**Keywords:** NAFLD, Early diagnosis, Metabolomics, LC–MS/MS, Biomarkers

## Abstract

**Background:**

Nonalcoholic fatty liver disease (NAFLD) is a common metabolic disease that affects 20–30% of individuals worldwide. Liver puncture remains the gold standard for the diagnosis of liver diseases despite limitations regarding invasive nature and sample variability. It is of great clinical significance to find noninvasive biomarkers to detect and predict NAFLD.

**Objective:**

The aims of this study were to identify potential serum markers in individuals with early-stage NAFLD and to advance the mechanistic understanding of this disease using a high-throughput mass spectrometry-based untargeted metabolomics approach.

**Methods:**

One hundred and twelve patients with early-stage NAFLD aged 18–55 were recruited according to the guidelines. The control group included 112 healthy participants. The demographic, anthropometric, clinical and laboratory data of all participants were systematically collected. Serum samples were obtained after an overnight fast. The comprehensive serum metabolomic analysis was performed by ultra-performance liquid chromatography-Orbitrap mass spectrometry. The resultant data was processed by Compound Discover and SIMCA-P software to validate the potential biomarkers. Significantly altered metabolites were evaluated by variable importance in projection value (*VIP* > 1) and ANOVA (*p* < 0.01). Pathway analysis was performed using MetaboAnalyst 4.0.

**Results:**

The liver function test of early NAFLD patients showed no statistical differences to control group (*p* > *0.05*). However, obvious differences in blood lipids were observed between subjects with NAFLD and controls (*p* < *0.001*). In total, 55 metabolites showed significant changes in experimental group were identified. The area under curve (AUC) values deduced by receiver operating curve (ROC) analysis indicated that these newly identified biomarkers have high predictability and reliability. Of these, 15 metabolites with AUC greater than 0.9 were of great diagnostic value in early NAFLD patients.

**Conclusion:**

In this study, a total of 15 serum metabolites were found to strongly associate with early NAFLD. These biomarkers may have great clinical significance in the early diagnosis of NAFLD, as well as to follow response to therapeutic interventions.

**Supplementary Information:**

The online version contains supplementary material available at 10.1186/s12967-021-02820-7.

## Background

In recent years, the trend in liver diseases has changed from traditional infectious diseases to metabolic disorders [1]. Liver disease is a high incidence disease in Asia [2–4]. There is strong evidence that a sedentary lifestyle and unhealthy dietary habits (especially those with high fat and high salt) are setting the stage for the prevalence of obesity and nonalcoholic fatty liver disease (NAFLD) in many urbanized Asian countries [5, 6]. NAFLD has also become a burgeoning health problem in developed country [2, 7]. The prevalence of NAFLD is highly underestimated because it often presents with minor to no symptoms in patients at the early stages. Given the increasing trends of obesity and metabolic syndrome, the two basic risk factors for NAFLD, incidence rates are expected to further rise in the next decades. Approximately 20–30% of patients with NAFLD progress to steatohepatitis and fibrosis that may progress to cirrhosis in extreme cases [8]. Considering the possible association between NAFLD-related cirrhosis and hepatocellular cancer (HCC), NAFLD is becoming an increasingly important problem in China where it is currently the primary/most common condition leading to cirrhosis) [9]. HCC has also been found to rise in patients with NAFLD in the absence of cirrhosis [10].

Currently, the histological examination of liver biopsy specimen remains the gold standard for NAFLD diagnosis despite well-acknowledged disadvantages, such as its invasive nature, inevitable sampling error, poor short-term repeatability and subjective differences among observers. Thus liver biopsy is unlikely to be carried out widely as a routine examination method in clinical practice [11]. Efficient diagnosis methods are needed for the facile identification of NAFLD patients, disease progression risk assessment, and monitoring the response to potential new treatment strategies. Radiologic technique including ultrasonography and magnetic resonance imaging (MRI) are the main representatives of non-invasive diagnostic modalities. The assessment of serum biomarkers that considered to be a more convenient and promising approaches for monitoring NAFLD has also been introduced [12, 13]. Alanine aminotransferase (ALT) and aspartate aminotransferase (AST) are the most common blood indicators; however, such liver enzymes may not elevate until histological injury of the liver occurs [14]. A number of patients with normal ALT levels may also have NAFLD and even advanced fibrosis. Therefore, the exploration and identification of novel biomarkers involved in the early stage of NAFLD is of crucial significance. As a high-throughput technology, metabolomics allows thousands of serum metabolites to be measured and identified simultaneously [15–17], and is ideally suited for the discovery of new biomarkers [18, 19]. In this study, the metabolic characteristics of blood samples from NAFLD patients were analyzed to explore more accurate and specific biomarkers.

## Materials and methods

### Chemicals and reagents

Methanol and acetonitrile (HPLC grade) were purchased from Fisher Chemicals (Waltham, USA); formic acid was purchased from Merck (Darmstadt, Germany); 2-chloro-DL-phenylalanine was from Merck (Darmstadt, Germany).

### Participants, inclusion & exclusion criteria

A total of 3802 participants who were hospitalized by the ward and outpatient of digestive department of Putuo Hospital Affiated to Shanghai University of Traditional Chinese Medicine were enrolled consecutively and separately between January 2019 and December 2020.

According to the guidelines for diagnosis and treatment of nonalcoholic fatty liver disease (2018) [20] formulated by the National Workship on Fatty Liver and Alcoholic Liver Disease and Chinese Society on Hepatology, the diagnosis of early-stage NAFLD was based on the detection of steatosis by abdominal ultrasonography. Only if three deputy director physicians make the same diagnosis could the patient be included.

The following exclusion criteria were used: (1) had a history of liver diseases other than NAFLD, including viral hepatitis, cirrhosis, liver cancer, autoimmune liver disease, alcoholic liver disease, hereditary liver disease, etc.; (2) excessive alcohol consumption (≥ 210 g/week for men, ≥ 140 g/week for women); (3) were taking medications that can affect metabolism or cause liver damage,; (4) had been diagnosed with diabetes, had received or were undergoing hypoglycemic drugs or insulin treatment; (5) had severe heart disease (myocardial infarction, heart failure and / or severe arrhythmia); (6) had severe infections and severe trauma; (7) pregnant or breastfeeding women; (8) had thyroid diseases, including hyperthyroidism and hypothyroidism**.**

**Fig. 1:**
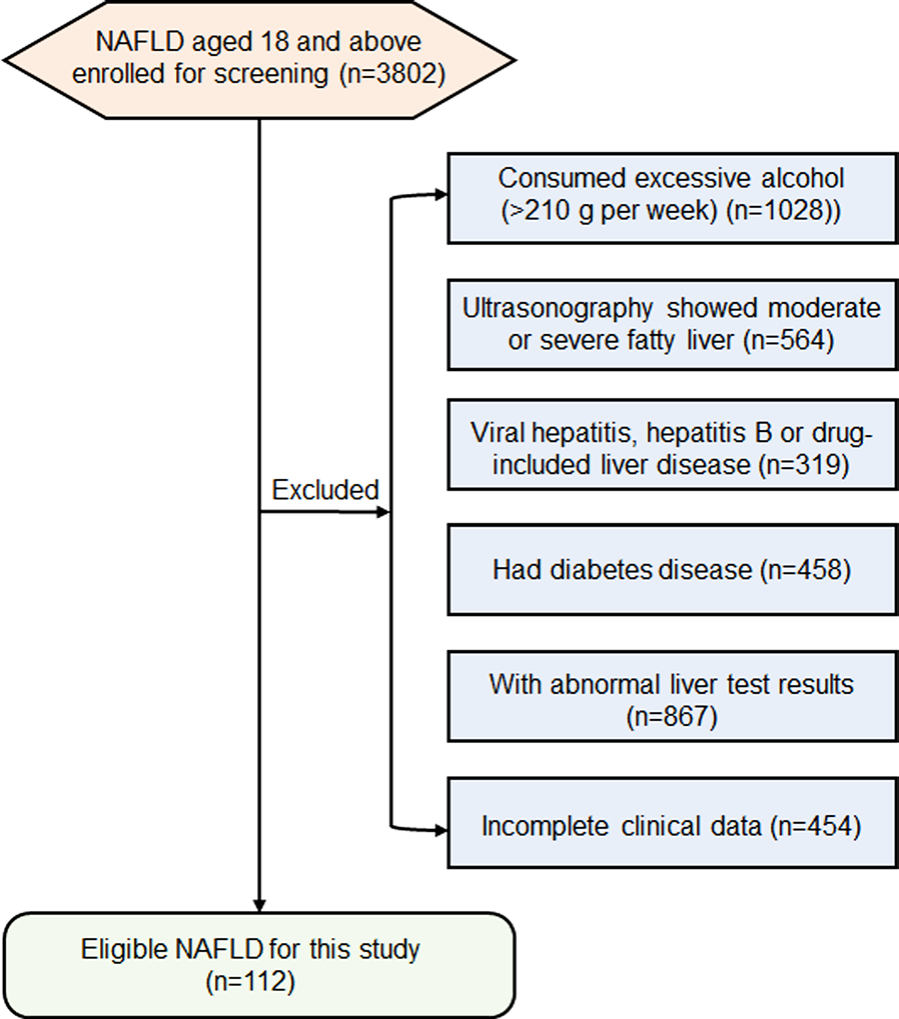
Flowchart of the study

In the end, 112 subjects (68 males and 44 females, aged ≥ 18 years) met the diagnosis criteria of the research (Fig. 1). The control group included 112 healthy people. This research was conducted in accordance with the Declaration of Helsinki to protect the health and rights of the participants. Written informed consent was obtained from each participant. The study protocol was approved by the Medical Ethics Committee of the Putuo Hospital Affiated to Shanghai University of traditional Chinese Medicine (Ethics approval number was PTEC-A-2018–49-1).

### Data collection

The medical history and physical examination of the participants were collected by the full-time doctors in the outpatient or ward of the department of gastroenterology, and the general conditions of the paticipants were recorded in detail, including name, gender, age, medical history, smoking and drinking history, etc. The total amount of alcohol consumed per week was calculated and classified into three grades: nondrinkers, light drinkers(< 70 g/week), or moderate drinkers (7–210 g/week for male and 70–140 g/week for female) and defined nondrinkers as did not drink any alcohol in the past 12 months [21].

### Anthropometric and biomedical measurements

The height, weight, waist circumference and blood pressure were measured in the morning by a specially assigned physician. The waist was measured at the middle point of the line between the lower edge of the arch and the iliac spine, measured twice for an average. Blood pressure was measured in quiet state for times, 10 min apart each time, and an average was taken by three times measurements. The body mass index (BMI) is a person's weight in kilograms divided by the square of height in meters.

After overnight fasting for 12 h, fasting blood was collected from the veins early in the morning. Measurements of blood glucose, ALT, AST, TB, DB, TP, ALB, Che, ALP, γ—GT, TG, TC, HDL-C, LDL-C and other serum indicators were performed on an automated chemistry analyzer (Hitachi 7600d-210, Japan).

### Sample preparation for metabolomics

One hundred microliters of serum was mixed with 400 μL methanol and 5 μL of 2-chloro-DL-phenylalanine (0.3 g/L, internal standard) for extraction. The supernatant of each sample was collected for LC–MS analysis after centrifugation at 15,000 rpm at 4 ℃ for 10 min.

### UPLC-Orbitrap MS condition

The serum metabolites profiling was performed on Ultimate 3000 UPLC system (Thermo Fisher Scientific) coupled with an Orbitrap Elite mass spectrometer (Thermo Fisher Scientific). Samples were eluted through an ACQUITY UPLC column (HSS T3, 100 × 2.1 mm, 1.8 μm, Waters Corp.) with a 20 min gradient (mobile phase A was 0.1% formic acid in water and mobile phase B was acetonitrile) at a flow rate of 300 nL/min. The solvent gradient was as follows: 0–2 min, 95% A; 2–12 min, 95–5% A; 12–15 min, 5% A; 15–20 min, 5–95% A.

The mass spectrometer was equipped with an electrospray ionization source and operated in both positive and negative ion modes. The source parameters were as follows: heater temperature, 300 °C; sheath gas flow, 45 psi; auxiliary gas flow, 5 L/min; tail gas flow, 0.3 L/min; electrospray voltage, 3.0 kV for positive ion mode and 3.2 kV for negative ion mode; capillary temperature, 350 °C; S-lens RF level, 30 and 60 for positive and negative ion mode, respectively.

### Data processing, biomarker identification and metabolomic pathway analysis

The raw LC–MS data were first processed with Compound Discover 2.0 software (Thermo Fisher Scientific). The Compound Discover software finds components that have reproducible differences across multiple sample groups. The resultant data matrix including *m/z*, RT and intensity was imported into the SIMCA-P 14.0 (Umetrics, Umea, Sweden) software for multivariate statistical analysis. PCA and OPLS-DA analyses were performed, and the variable importance projection (VIP) value was used to screen potential biomarkers. Metabolites of interest (candidate biomarkers) were identified based on their accurate masses and/or MS/MS spectra information in both positive and negative ion mode. HMDB, KEGG and mzCloud databases were searched to assist with metabolite identification. Pathway analysis of the significant altered metabolites was performed with MetaboAnalyst 4.0.

### Statistical analysis

SPSS 25.0 software (Chicago, United States) was used to perform two-way ANOVAs, receiver operating characteristic (ROC) curve and logistic regression analyses among two groups. The area under the ROC curve (AUC) was used to evaluate the diagnostic power of each potential biomarker in NAFLD. Data were presented as percentages for categorical variables and as means ± SD for continuous variables. Differences in categorical and continuous variables between groups were assessed with the *χ*^2^ test and the independent samples t-test, while the non-normal distribution was expressed by M (p25-p75), the Wilcoxon nonparametric test was used between the two groups. In all cases, *p* < 0.05 was considered as significant.

## Results

### Demographic characteristics

From Table 1, we found that among the 224 subjects, the NAFLD group had significant differences in body weight, BMI, and waist statistics compared with the control group (*p* < 0.001), while there were no obvious differences in age, gender, height, systolic blood pressure, diastole blood pressure, smoking and drinking (*p* > 0.05).

**Table 1:** Clinical and serum biochemical parameters of subjects

	NAFLD (n = 112)	Control (n = 112)	*P* value
Gender (M/F)	68/44	62/50	
Age	46 ± 14	41 ± 15	0.538
Height (cm)	169.42 ± 8.73	170.59 ± 8.07	0.615
Weight (kg)	77.55 ± 13.49	62.37 ± 8.05	0.001**
BMI (kg/m^2^)	27.1(24–28.55)	21.46(20.57–22.54)	0.001**
Systolic pressure (mmHg)	125.41(120–130)	123.59(115–132)	0.375
Diastolic pressure (mmHg)	79.74(75–85)	77.63(74–81)	0.183
Waist circumference (cm)	91.75 ± 11.76	72.04 ± 5.96	0.001
Heart rate (BPM)	68.68 ± 5.09	68.80 ± 4.88	0.851
Smoking (n)	62	83	0.154
Drinking (n)			0.916
None	10	6	
Light	38	43	
Moderate	64	63	
Blood glucose (mmol/L)	5.12 ± 0.35	5.21 ± 0.42	0.38
TB (umol/L)	12.74 ± 2.54	12.89 ± 3.62	0.862
DB (umol/L)	2.32 ± 0.5	2.23 ± 0.95	0.669
TP (g/L)	73.52 ± 4.71	73.11 ± 5.4	0.769
ALB (umol/L)	41.85 ± 2.99	41.37 ± 5.46	0.689
CHE (U/L)	8400.78 ± 1353.66	7694.52 ± 1640.95	0.09
ALT (U/L)	20.67 ± 11.67	28.04 ± 19.13	0.093
AST (U/L)	23.93 ± 6.49	27.7 ± 13.02	0.183
ALP (U/L)	76.96 ± 18.08	84.33 ± 22.15	0.186
γ-GT(U/L)	23.74 ± 11.8	31.59 ± 22.75	0.118
TC (mmol/L)	5.67 ± 1.22	4.57 ± 0.8	0.001**
TG (mmol/L)	0.83 ± 0.96	1.24 ± 0.56	0.008*
HDL-C (mmol/L)	0.98 ± 0.18	1.49 ± 0.35	0.002*
LDL-C (mmol/L)	4.89 ± 0.73	3.65 ± 1.01	0.002*

### Serum index

From Table 1, we found that there was no remarkable difference in biochemical indexes (including TBA, CHE, TB, DB, TP, ALB, γ-GT, ALP, AST, ALT and GLU) between the NAFLD group and the control group (*p* > 0.05). However, conspicuous differences in all blood lipid indexes (HDL, LDL, TC and TG) between the two groups (*p* < 0.001, *p* < 0.01, *p* < 0.01, *p* < 0.01) were observed.

### PCA of serum samples in NAFLDs

**Fig.2:**
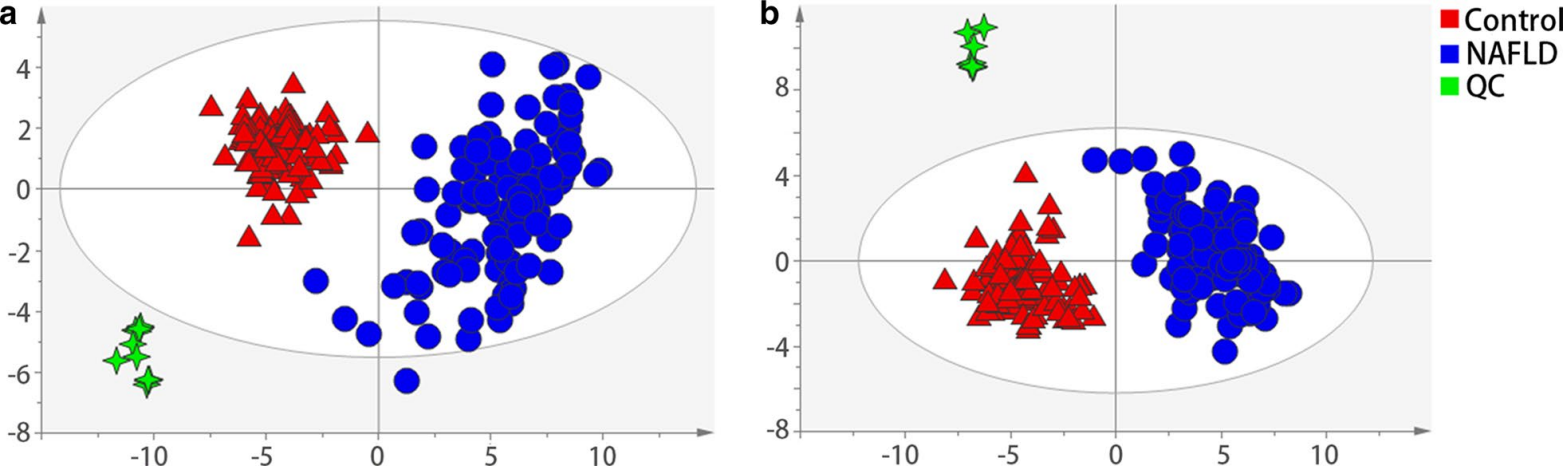
The PCA score plots of serum samples from control group and NAFLDs in (A) positive ion mode and (B) negative ion mode (Control, n = 112; NAFLD, n = 112)

PCA was performed for both positive and negative ionization modes. Quality control samples were determined for instrument precision assessments and the results confirmed the stability of the UPLC-MS/MS system. As can been seen in Fig. 2, NAFLDs compared to controls showed distinct separations in the PCA score plots, indicating global changes to serum metabolite composition in NAFLD. The cumulative values of R^2^X and Q^2^Y in both ionization modes suggested the excellent classification and prediction ability of the model.

### OPLS-DA and metabolites identification in serum from NAFLD patients

OPLS-DA was employed in NAFLD and control groups to identify potential metabolic biomarkers. The R^2^Y and Q^2^Y values were 0.986 and 0.895 in positive ion mode, respectively, and 0.957 and 0.877 in negative ion mode, respectively. The data indicated that the degree of the method’s discrimination and predictability met the analysis requirements. The VIP and *p*-values were used to screen potential metabolic biomarkers. Fifty-five metabolites in serum met the retrieval requirements (*VIP* > 1 and *p* < 0.01) and were identified by using the databases (Table 2).

**Table 2:** Efficiency comparison of diagnostic indicators

No	Name	AUC	95% CI		Sensitivity	Specificity
			Lower limit	Upper limit		
1	LysoPC(20:3(8Z,11Z,14Z))	0.97	0.933	1	85.19	100.00
2	Succinic acid	0.963	0.917	1	100.00	88.89
3	LysoPC(22:5(7Z,10Z,13Z,16Z,19Z))	0.949	0.896	1	96.30	85.19
4	Indole	0.947	0.881	1	88.89	96.30
5	LysoPC(22:4(7Z,10Z,13Z,16Z))	0.942	0.887	0.998	81.48	92.59
6	Oleic acid	0.938	0.878	0.998	85.19	92.59
7	Desaminotyrosine	0.926	0.849	1	81.48	92.59
8	L-Phenylalanine	0.918	0.838	0.997	92.59	81.48
9	L-Tryptophan	0.915	0.825	1	85.19	96.30
10	LysoPE(22:2(13Z,16Z)/0:0)	0.915	0.837	0.993	85.19	85.19
11	Leukotriene C5	0.909	0.831	0.988	92.59	81.48
12	1-Alkyl-2-acylglycerophosphoethanolamine	0.905	0.829	0.981	81.48	81.48
13	LysoPE(0:0/20:3(5Z,8Z,11Z))	0.905	0.83	0.981	77.78	88.89
14	L-Lysine	0.905	0.815	0.995	85.19	88.89
15	LysoPE(0:0/22:4(7Z,10Z,13Z,16Z))	0.9	0.82	0.979	96.30	70.37
16	Homovanillic acid	0.9	0.801	0.999	88.89	96.30
17	Sulfuric acid	0.894	0.81	0.979	77.78	88.89
18	LysoPE(16:1(9Z)/0:0)	0.893	0.806	0.98	92.59	74.07
19	Coumarone	0.886	0.797	0.975	77.78	88.89
20	LysoPC(22:6(4Z,7Z,10Z,13Z,16Z,19Z))	0.885	0.796	0.974	77.78	88.89
21	LysoPC(20:4(8Z,11Z,14Z,17Z))	0.879	0.784	0.975	81.48	85.19
22	LysoPE(0:0/22:5(4Z,7Z,10Z,13Z,16Z))	0.878	0.78	0.976	74.07	92.59
23	Benzoic acid	0.877	0.784	0.969	77.78	88.89
24	LysoPC (20:2(11Z,14Z))	0.87	0.768	0.972	74.07	100.00
25	LysoPE(0:0/24:6(6Z,9Z,12Z,15Z,18Z,21Z))	0.87	0.761	0.978	85.19	81.48
26	LysoPC (20:4(5Z,8Z,11Z,14Z))	0.868	0.771	0.965	81.48	85.19
27	1-arachidonoyl-sn-glycero-3-phosphoethanolamine	0.867	0.774	0.96	88.89	70.37
28	1-Acyl-sn-glycero-3-phosphoethanolamine	0.863	0.745	0.98	74.07	96.30
29	L-methionine	0.86	0.758	0.963	85.19	77.78
30	4-Hydroxycinnamic acid	0.855	0.753	0.956	70.37	92.59
31	Arachidonic acid	0.85	0.75	0.951	85.19	70.37
32	L-TYROSINE	0.846	0.741	0.952	70.37	92.59
33	L-Lactic Acid	0.845	0.738	0.952	92.59	66.67
34	Palmitic Acid	0.844	0.72	0.967	85.19	88.89
35	LysoPE(20:1(11Z)/0:0)	0.841	0.73	0.952	85.19	77.78
36	LysoPC(18:2(9Z,12Z))	0.833	0.724	0.941	70.37	92.59
37	LysoPC(18:1(9Z))	0.826	0.711	0.94	70.37	96.30
38	Ethyl acetate	0.824	0.709	0.94	74.07	88.89
39	1-[(9Z)-hexadecenoyl]-sn-glycero-3-phosphocholine	0.822	0.708	0.935	96.30	59.26
40	LysoPC (15:0)	0.82	0.694	0.947	77.78	92.59
41	Glycerylphosphorylcholine	0.818	0.688	0.947	77.78	92.59
42	LysoPC(18:3(9Z,12Z,15Z))	0.816	0.706	0.926	74.07	77.78
43	Uric Acid	0.815	0.69	0.939	66.67	92.59
44	2-Acyl-sn-glycero-3-phosphoethanolamine	0.811	0.694	0.928	81.48	70.37
45	Stearic acid	0.793	0.664	0.922	70.37	85.19
46	LysoPE (0:0/20:0)	0.785	0.65	0.92	77.78	74.07
47	L-Palmitoylcarnitine	0.761	0.628	0.895	70.37	85.19
48	LysoPE(0:0/16:0)	0.76	0.626	0.894	77.78	66.67
49	2-linoleoyl-sn-glycero-3-phosphoethanolamine	0.749	0.618	0.88	66.67	77.78
50	Platelet-activating factor	0.708	0.563	0.852	51.85	96.30
51	L-Carnitine	0.705	0.568	0.842	59.26	74.07
52	1-heptadecanoyl-sn-glycero-3-phosphocholine	0.672	0.52	0.824	55.56	85.19
53	L-Valine	0.236	0.102	0.37	14.81	33.33
54	L-Pyroglutamic acid	0.091	0.007	0.174	22.22	7.41
55	Citric acid	0.056	0	0.131	3.70	7.41

### Pathway enrichment analysis

**Fig.3:**
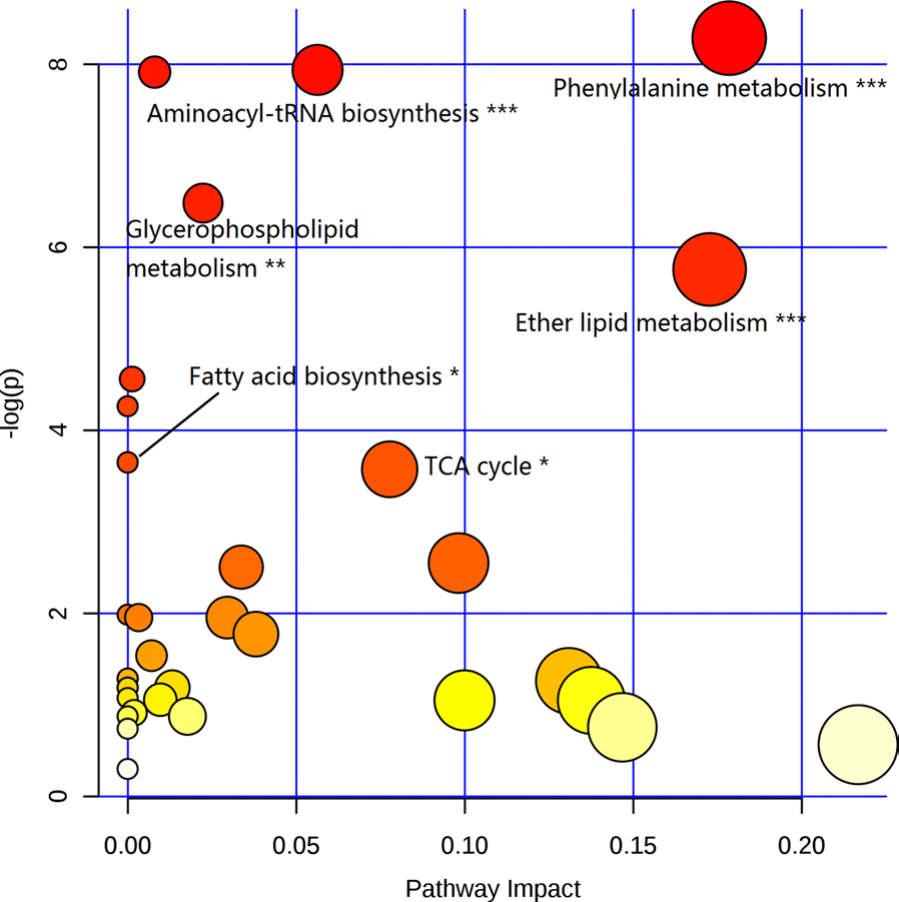
Pathway analysis of significant altered metabolites

Pathway enrichment was achieved using MetaboAnalyst 4.0 and the results are showed in Fig. 3. The pathways with significant differences (*p* < 0.05) are: (1) phenylalanine metabolism; (2) aminoacyl-tRNA biosynthesis; (3) glycerophospholipid metabolism; (4) ether lipid metabolism; (5) fatty acid biosynthesis; and (6) the tricarboxylic acid cycle (TCA cycle).

### Diagnostic accuracy of the candidate biomarkers for early NAFLD

**Fig.4:**
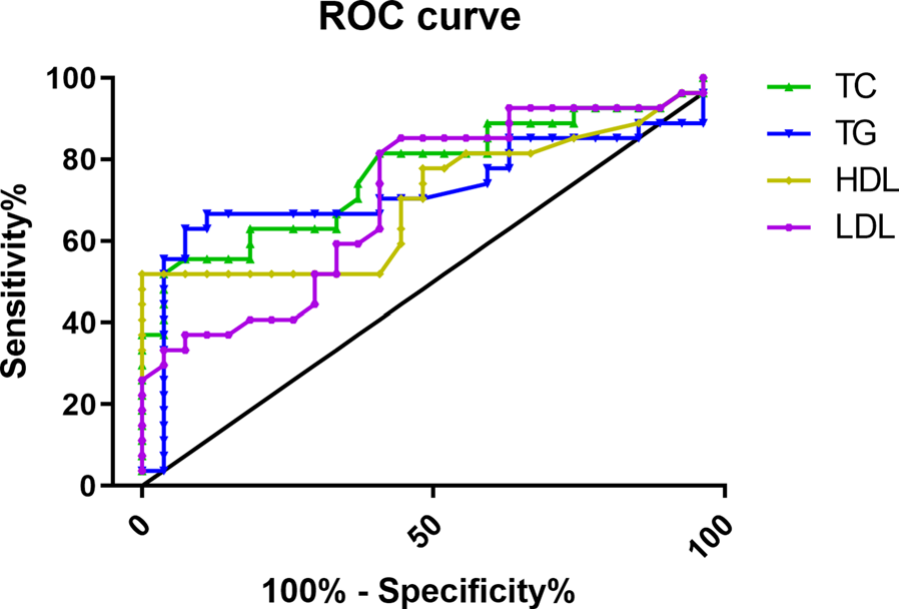
ROC curve of clinical indicators

**Fig.5:**
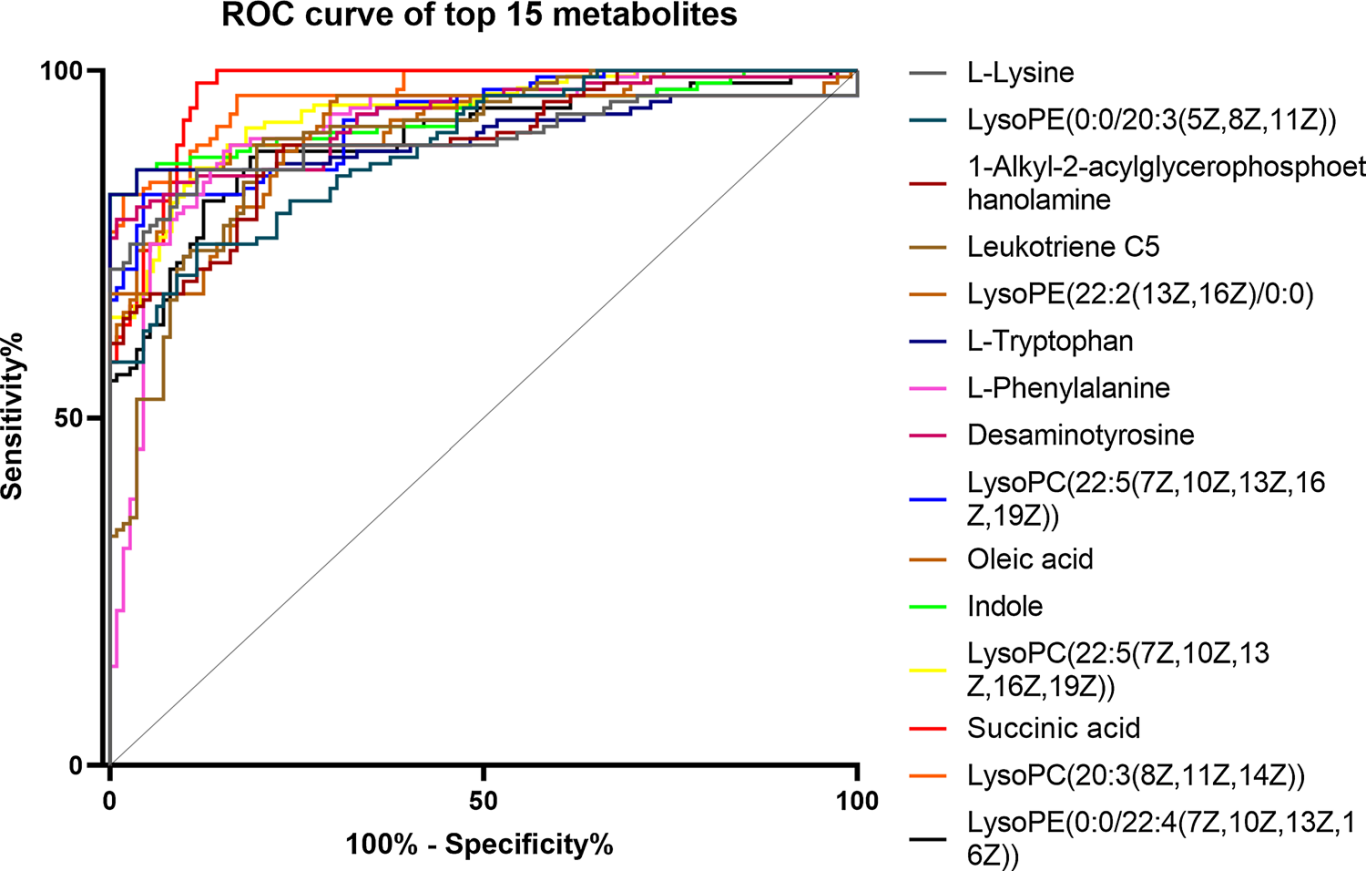
ROC curve of the new biomarkers

The ROC curves of the four lipids (TC, TG, HDL and LDL) were 0.770, 0.728, 0.706 and 0.711, respectively (Fig. 4). A detailed summary of the AUCs, 95% CI lower and upper limit, sensitivities and specificities of the identified serum metabolites are shown in Table 2. The AUCs for 15 metabolites in serum were above 0.9, indicating that they were of high diagnostic value (Fig. 5).

## Discussion

Nowadays, NAFLD has risen as the most common chronic liver disease in China [22, 23]. Many studies have shown that the prevalence of NAFLD increases in parallel with components of the metabolic syndrome such as obesity, type 2 diabetes mellitus (T2DM), hyperlipidemia and hypertension [24, 25].The prevalence of NAFLD in industrialized countries is considered to be between 40 and 50%,even higher in patients with T2DM, and the prevalence is up to 90% in morbidly obese patients [26, 27]. The relation between NAFLD and T2DM is considered bidirectional. In addition to the evidence that insulin resistance may contribute to progressive liver disease, NAFLD may also predispose to developing or worsening insulin resistance and metabolic syndrome [28]. Although the natural history of NAFLD is not fully understood, participants with T2DM were excluded in this study.

In addition to the type and frequency of alcohol consumed, it is also unsettled whether moderate alcohol intake plays a role in the development of NAFLD. Many studies suggested that alcohol consumption below safe limits can promote lipid metabolism and reduce insulin resistance, thereby reducing the prevalence of NAFLD, whereas others have reported deterioration of steatohepatitis and fibrosis [29–31]. Nondrinkers and subjects with moderate alcohol consumption that compatible with the diagnosis of NAFLD were included in this study. No clear association between NAFLD and the total alcohol intake per week was observed.

Liver enzymes, especially ALT and AST, are the first laboratory tests every clinician will consider worth evaluating in a patient with liver diseases.In most cases, the higher the ALT and AST levels, the more severe the liver damage [32]. However, all patients included in this study were early NAFLD patients showed normal liver chemistry, thus there were no statistically significant differences in serum concentrations of ALT, AST, ALP, γ-GT, TB, DB, TP, ALB and CHE between the two groups (Additional file 1: Fig. S1).

In the current study, we found that TG, TC, LDL-C and HDL-C were closely related to NAFLD disease. NAFLD patients tend to have high TG, high TC, high LDL-C and low HDL-C levels which in consistence with a previous study reported by Malik and coworkers [33]. However, Abdul et al*.* [34] found that there was no obvious relationship between NAFLD and LDL-C, TC, but a significant relationship with TG. In addition, Fang [35] used the TG / HDL-C ratio as a predictor of NAFLD. From this, it can be found that whether lipid level can be used as a predictor of the progression of NAFLD is still controversial.

It has been reported that liver lipotoxicity of free fatty acids, cholesterol, ceramide and lysophosphatidylcholine is the main reason for the progression of NAFLD. The simple accumulation of triglycerides may not lead to NAFLD [36–38], but rather, the type of accumulated lipids may determine the severity and development trend of NAFLD. The results of metabolomic analysis showed that the levels of lysoPC, lysoPE, phenylalanine, oleic acid and tryptophan were obviously increased in NAFLD patients with hepatitis [39–42]. These findings suggested that these serum metabolites play important roles in the development of NAFLD, as well as their considerable clinical value. An issue that was not addressed in the present study was the lead-time bias. Since there is a period of time before the detection of NAFLD in which NAFLD has developed with no clinical manifestations, the effectiveness of these biomarkers might be changed whenever estimated during this period.

An overview of the altered pathways is shown in Fig. 3. The contents of all metabolites in phenylalanine metabolism pathway were increased. Phenylalanine and its related metabolites are mainly metabolized in the liver [43–45]. Some studies have showed that the increased levels of phenylalanine are highly correlated with obesity and liver steatosis [46–48]. Other studies have found that phenylalanine levels in T2DM patients are significantly increased, especially after a normal diet [49]. In addition, through logistic regression analysis from 72 high and 75 low insulin sensitivity subjects, Palmer et al*.* [50] observed significantly decreased glycine and increased valine, leucine, phenylalanine, and combined glutamine and glutamate in insulin-resistant subjects. Therefore, deterioration of liver function in NAFLD patients may cause the decline in phenylalanine metabolism, and ultimately lead to the accumulation of phenylalanine and its related metabolites in liver and serum.

## Conclusion

By high-throughput LC–MS-based metabolomics analysis, a total of 55 metabolites significantly associate with early-stage NAFLD were identified. Of these, 15 potential biomarkers showed high diagnosis value with AUC greater than 0.9. Moreover, our results provide comprehensive insights into the metabolic pathways involved in NAFLD. These combined serum metabolites could be the basis of a clinically feasible method of non-invasive NAFLD screening, as well as to follow response to therapeutic interventions.

## Supplementary Information

**Additional file 1**: **Fig. S1**. Liver images of early fatty liver disease.

## Data Availability

All data are included in this article.

## Abbreviations

NAFLD: Nonalcoholic Fatty Liver Disease
TBA: Total bile acid
CHE: Cholinesterase
TB: Total Bilirubin
DB: Direct Bilirubin
TP: Total Protein
ALB: Albumin
γ-GT: γ-Glutamyltransferase
ALP: Alkaline Phosphatase
AST: Aspartate Aminotransferase
ALT: Alanine Aminotransferase
HDL: High Density Lipoprotein
LDL: Low Density Lipoprotein
TC: Cholesterol
TG: Triglyceride
GLU: Glucose in Urine
PCA: Principal Component Analysis
OPLS-DA: Orthogonal Partial Least Squares Discriminant Analysis
VIP: Variable Importance in Projection
LC–MS: Liquid Chromatography Mass Spectrometry
AUC: Area under Curve
ROC: Receiver Operating Curve
