# Correction to: Metabolic analysis of early nonalcoholic fatty liver disease in humans using liquid chromatography–mass spectrometry

**DOI:** 10.1186/s12967-021-03223-4

**Published:** 2022-01-10

**Authors:** Cheng Hu, Tao Wang, Xiaoyu Zhuang, Qiaoli Sun, Xiaochun Wang, Hui Lin, Mingli Feng, Jiaqi Zhang, Qin Cao, Yuanye Jiang

**Affiliations:** 1 Experiment Center for Science and Technology, Shanghai University of Traditional Chinese Medicine, Shanghai, 201203 China; 2 Department of Gastroenterology, Putuo Hospital, Shanghai University of Traditional Chinese Medicine, 164 Lanxi Road, Shanghai, 200062 China; 3 Institute of Digestive Diseases, Longhua Hospital, Shanghai University of Traditional Chinese Medicine, Shanghai, 200032 China; 4 Shanghai TCM-Integrated Institute of Vascular Anomalies, Shanghai TCM-Integrated Hospital, Shanghai University of Traditional Chinese Medicine, Shanghai, 200082 China

## Correction to: J Transl Med (2021)19:152 https://doi.org/10.1186/s12967-021-02820-7

Following publication of the original article [1], the authors identified an error in the affiliation for Jiaqi Zhang. The incorrect and correct affiliation information is shown below. The author group has been updated above and the original article [1] has been corrected.

Incorrect:

^4^Shanghai TCM-Integrated Institute of Vascular Anomalies, Shanghai TCM-Integrated Hospital, Shanghai University of Traditional Chinese Medicine, Shanghai 200082, China.

Correct:

^1^Experiment Center for Science and Technology, Shanghai University of Traditional Chinese Medicine, Shanghai 201203, China.

^4^Shanghai TCM-Integrated Institute of Vascular Anomalies, Shanghai TCM-Integrated Hospital, Shanghai University of Traditional Chinese Medicine, Shanghai 200082, China.
