# Supplementary figures and images for: Metabolic analysis of early nonalcoholic fatty liver disease in humans using liquid chromatography-mass spectrometry
Source: J Transl Med. 2021 Apr 15;19:152. doi: 10.1186/s12967-021-02820-7 (PMC8050915; doi:10.1186/s12967-021-02820-7)

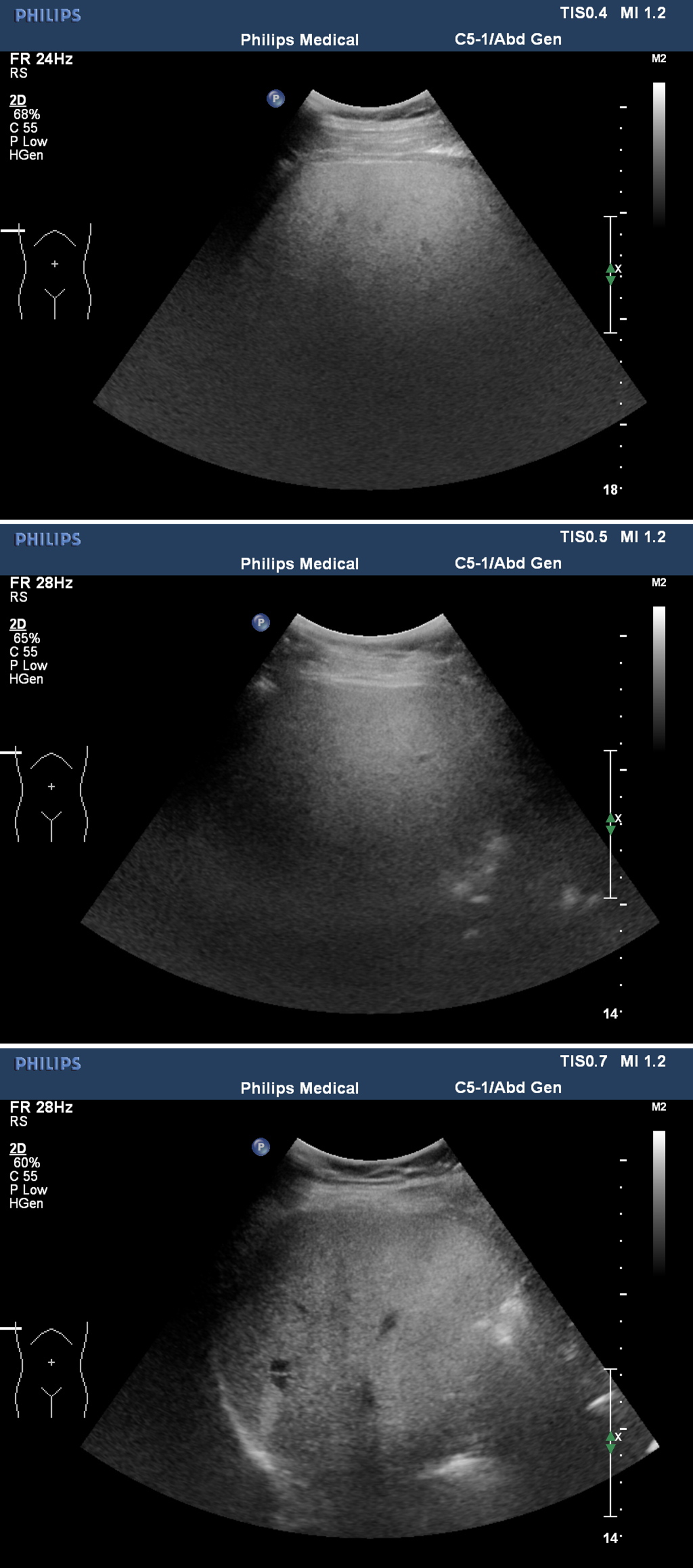

Supplement: Supplementary file 1 — Additional file 1: Fig. S1. Liver images of early fatty liver disease. [file 12967_2021_2820_MOESM1_ESM.tif]
